# 4-[(4-Amino­phen­yl)sulfon­yl]aniline–3,5-dinitro­benzoic acid (1/1)

**DOI:** 10.1107/S1600536812004709

**Published:** 2012-02-10

**Authors:** Graham Smith, Urs D. Wermuth

**Affiliations:** a Science and Engineering Faculty, Queensland University of Technology, GPO Box 2434, Brisbane, Queensland 4001, Australia

## Abstract

The title compound, C_7_H_4_N_2_O_6_·C_12_H_12_N_2_O_2_S, is a 1:1 cocrystal of the drug dapsone with 3,5-dinitro­benzoic acid. The dihedral angle between the two aromatic rings of the dapsone mol­ecule is 75.4 (2)°, and the dihedral angles between these rings and that of the 3,5-dinitro­benzoic acid are 64.5 (2) and 68.4 (2)°. A strong inter­molecular carb­oxy­lic acid O—H⋯N_amine_ hydrogen bond is found, together with inter­molecular amine N—H⋯O hydrogen-bonding associations with carboxyl, nitro and sulfone O-atom acceptors. In addition, weak π–π inter­actions between one of the dapsone benzene rings and the 3,5-dinitro­benzoic acid ring [ring centroid separation = 3.774 (2) Å] results in a two-dimensional network structure.

## Related literature

For drug applications of dapsone, see: Wilson *et al.* (1991 ▶). For the structures of dapsone and its salts and adducts, see: Dickenson *et al.* (1970 ▶); Kus’mina *et al.* (1981 ▶); Smith & Wermuth (2012*a* ▶,*b* ▶). For adducts of 3,5-dinitro­benzoic acid, see: Etter & Frankenbach (1989 ▶).
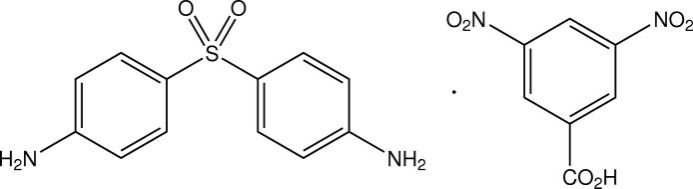


## Experimental

### 

#### Crystal data

C_7_H_4_N_2_O_6_·C_12_H_12_N_2_O_2_S
*M* *_r_* = 460.43
Monoclinic, 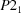

*a* = 5.8222 (4) Å
*b* = 15.5982 (10) Å
*c* = 10.7299 (9) Å
β = 97.693 (6)°
*V* = 965.68 (12) Å^3^
*Z* = 2
Mo *K*α radiation
μ = 0.23 mm^−1^
*T* = 200 K
0.30 × 0.25 × 0.05 mm

#### Data collection

Oxfod Diffraction Gemini-S CCD detector diffractometer
Absorption correction: multi-scan (*CrysAlis PRO*; Oxford Diffraction, 2010 ▶) *T*_min_ = 0.832, *T*_max_ = 0.990
6257 measured reflections
3774 independent reflections
2643 reflections with *I* > 2σ(*I*)
*R*_int_ = 0.049

#### Refinement

*R*[*F* ^2^ > 2σ(*F* ^2^)] = 0.056
*wR*(*F* ^2^) = 0.102
*S* = 0.93
3774 reflections
289 parameters
1 restraint
H-atom parameters constrained
Δρ_max_ = 0.50 e Å^−3^
Δρ_min_ = −0.43 e Å^−3^
Absolute structure: Flack (1983 ▶), 1803 Friedel pairs
Flack parameter: 0.07 (11)

### 

Data collection: *CrysAlis PRO* (Oxford Diffraction, 2010 ▶); cell refinement: *CrysAlis PRO*; data reduction: *CrysAlis PRO*; program(s) used to solve structure: *SIR92* (Altomare *et al.*, 1994 ▶); program(s) used to refine structure: *SHELXL97* (Sheldrick, 2008 ▶) within *WinGX* (Farrugia, 1999 ▶); molecular graphics: *PLATON* (Spek, 2009 ▶); software used to prepare material for publication: *PLATON*.

## Supplementary Material

Crystal structure: contains datablock(s) global, I. DOI: 10.1107/S1600536812004709/fj2510sup1.cif

Structure factors: contains datablock(s) I. DOI: 10.1107/S1600536812004709/fj2510Isup2.hkl

Supplementary material file. DOI: 10.1107/S1600536812004709/fj2510Isup3.cml

Additional supplementary materials:  crystallographic information; 3D view; checkCIF report

## Figures and Tables

**Table 1:** Hydrogen-bond geometry (Å, °)

*D*—H⋯*A*	*D*—H	H⋯*A*	*D*⋯*A*	*D*—H⋯*A*
O12*A*—H12*A*⋯N41	0.93	1.73	2.653 (5)	173
N4—H412⋯O31*A*^i^	0.95	2.49	3.150 (5)	126
N41—H413⋯O11*A*^ii^	0.89	2.50	3.367 (5)	165

Symmetry codes: (i) 
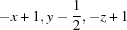
; (ii) 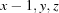.

**Fig. 1.**
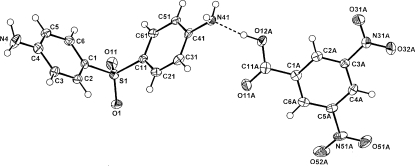
The molecular conformation and atom-numbering scheme for the Dapsone and 3,5-dinitrobenzoic acid molecules in the title co-crystal. Non-H atoms are shown as 50% probability displacement ellipsoids and the inter-species hydrogen bond is shown as a dashed line.

**Fig. 2.**
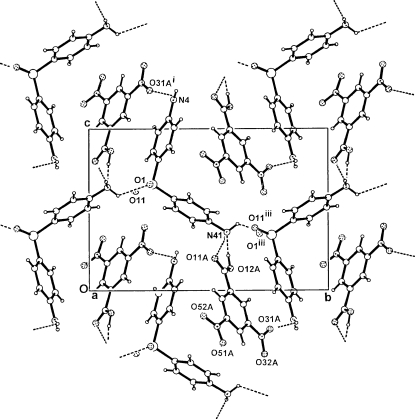
The hydrogen-bonding in the title adduct, viewed down the a axial direction of the unit cell. Hydrogen bonds are shown as dashed lines. For symmetry codes see Table 1.

## supplementary crystallographic information

### Comment

Dapsone [4-(4-aminophenylsulfonyl)aniline] is a very weak Lewis base which finds use as an anti-leprotic, anti-malarial and leprostatic drug (Wilson *et al.*, 1991). The structure of the Dapsone 0.33hydrate is known (Kus'mina *et al.*, 1981) but salts or adducts of this compound are not common. We have reported the 1:2 co-crystalline adduct with 1,3,5-trinitrobenzene (Smith & Wermuth, 2012*a*). Reported here is the structure of the 1:1 cocrystalline adduct of Dapsone with 3,5-dinitrobenzoic acid, C_12_H_12_N_2_O_2_S. C_7_H_4_N_4_O_6_ (Fig. 1). This acid has been found to be very useful for the formation of co-crystalline adducts (Etter & Frankenbach, 1989).

A primary intermolecular *O*—*H*···O_amine_ hydrogen bond (Table 1) links the two molecules while N—H···O hydrogen-bond associations with carboxyl, nitro and sulfone O-atom acceptors give a two-dimensional structure (Fig. 2). A weak π–π interaction is also found between one of the Dapsone aromatic ring moieties (C1–C6) and that of the acid molecule (C1*A*–C6*A* [minimum ring centroid separation 3.774 (2) Å]. In the Dapsone molecule the inter-ring dihedral angle is 75.4 (2)° which compare with 77.3° in the anhydrous parent Dapsone molecule (Dickenson *et al.*, 1970), 88.1, 75.8 and 74.7° for the three independent Dapsone molecules in the 0.33hydrate structure (Kus'mina *et al.*, 1981) and 77.5° in the 5-nitroisophthalic acid adduct (Smith & Wermuth, 2012*b*. The 3,5-dinitrobenzoic acid molecule is essentially planar [torsion angles C2*A*—C1*A*—C11*A*—O11*A*, -171.3 (4)°; C2*A*—C3*A*—N31*A*—O32*A*, -174.4 (4)°; C4*A*—C5*A*—N51*A*—O52*A*, -172.2 (4)°].

### Experimental

The title compound was prepared by the intereaction of 4-(4-aminophenylsulfonyl)aniline (Dapsone) with 3,5-dinitrobenzoic acid by heating together for 15 min under reflux, 1 mmol quantities of the two reagents in 50 ml of 50% ethanol–water. Minor poorly-formed yellow crystal aggregates of the title co-crystal formed after partial room-temperature evaporation of the solvent.

### Refinement

All H atoms potentially involved in hydrogen-bonding associations were located in a difference-Fourier analysis but were subsequently constrained, with *U*_iso_(H) = 1.2*U*_eq_(N, O). Other H-atoms were included at calculated positions [C—H = 0.93 Å] and also treated as riding, with *U*_iso_(H) = 1.2*U*_eq_(C). No reasonable acceptor atom could be found for one of the amine H-atoms on N4 (H411).

### Crystal data

**Table:**

C_7_H_4_N_2_O_6_·C_12_H_12_N_2_O_2_S	*F*(000) = 476
*M**_r_* = 460.43	*D*_x_ = 1.584 Mg m^−^^3^
Monoclinic, *P*2_1_	Mo *K*α radiation, λ = 0.71073 Å
Hall symbol: P 2yb	Cell parameters from 2802 reflections
*a* = 5.8222 (4) Å	θ = 3.2–28.7°
*b* = 15.5982 (10) Å	µ = 0.23 mm^−^^1^
*c* = 10.7299 (9) Å	*T* = 200 K
β = 97.693 (6)°	Plate, yellow
*V* = 965.68 (12) Å^3^	0.30 × 0.25 × 0.05 mm
*Z* = 2	

### Data collection

**Table:**

Oxfod Diffraction Gemini-S CCD detector diffractometer	3774 independent reflections
Radiation source: Enhance (Mo) X-ray source	2643 reflections with *I* > 2σ(*I*)
Graphite monochromator	*R*_int_ = 0.049
Detector resolution: 16.077 pixels mm^-1^	θ_max_ = 26.0°, θ_min_ = 3.2°
ω scans	*h* = −7→7
Absorption correction: multi-scan (*CrysAlis PRO*; Oxford Diffraction, 2010)	*k* = −19→19
*T*_min_ = 0.832, *T*_max_ = 0.990	*l* = −13→10
6257 measured reflections	

### Refinement

**Table:**

Refinement on *F*^2^	Secondary atom site location: difference Fourier map
Least-squares matrix: full	Hydrogen site location: inferred from neighbouring sites
*R*[*F*^2^ > 2σ(*F*^2^)] = 0.056	H-atom parameters constrained
*wR*(*F*^2^) = 0.102	*w* = 1/[σ^2^(*F*_o_^2^) + (0.0468*P*)^2^] where *P* = (*F*_o_^2^ + 2*F*_c_^2^)/3
*S* = 0.93	(Δ/σ)_max_ = 0.001
3774 reflections	Δρ_max_ = 0.50 e Å^−^^3^
289 parameters	Δρ_min_ = −0.43 e Å^−^^3^
1 restraint	Absolute structure: Flack (1983), 1803 Friedel pairs
Primary atom site location: structure-invariant direct methods	Flack parameter: 0.07 (11)

### Special details

**Table:**

Geometry. Bond distances, angles *etc*. have been calculated using the rounded fractional coordinates. All su's are estimated from the variances of the (full) variance-covariance matrix. The cell e.s.d.'s are taken into account in the estimation of distances, angles and torsion angles
Refinement. Refinement of *F*^2^ against ALL reflections. The weighted *R*-factor *wR* and goodness of fit *S* are based on *F*^2^, conventional *R*-factors *R* are based on *F*, with *F* set to zero for negative *F*^2^. The threshold expression of *F*^2^ > σ(*F*^2^) is used only for calculating *R*-factors(gt) *etc*. and is not relevant to the choice of reflections for refinement. *R*-factors based on *F*^2^ are statistically about twice as large as those based on *F*, and *R*- factors based on ALL data will be even larger.

### Fractional atomic coordinates and isotropic or equivalent isotropic displacement parameters (Å^2^)

**Table:**

	*x*	*y*	*z*	*U*_iso_*/*U*_eq_	
S1	0.93142 (16)	0.26625 (7)	0.65868 (10)	0.0211 (3)	
O1	1.1787 (4)	0.25679 (19)	0.6593 (2)	0.0277 (9)	
O11	0.7823 (5)	0.19588 (17)	0.6150 (3)	0.0281 (10)	
N4	0.8049 (6)	0.3557 (2)	1.1801 (3)	0.0372 (14)	
N41	0.6306 (6)	0.5772 (2)	0.3593 (3)	0.0297 (12)	
C1	0.8876 (6)	0.2919 (2)	0.8133 (4)	0.0198 (12)	
C2	1.0600 (7)	0.3366 (2)	0.8888 (4)	0.0214 (14)	
C3	1.0320 (7)	0.3575 (3)	1.0091 (4)	0.0234 (14)	
C4	0.8282 (7)	0.3357 (3)	1.0594 (4)	0.0244 (14)	
C5	0.6570 (6)	0.2913 (2)	0.9813 (4)	0.0250 (16)	
C6	0.6843 (6)	0.2705 (3)	0.8600 (4)	0.0232 (14)	
C11	0.8420 (6)	0.3568 (2)	0.5669 (4)	0.0179 (12)	
C21	0.9984 (6)	0.4232 (2)	0.5568 (4)	0.0216 (14)	
C31	0.9285 (7)	0.4947 (3)	0.4881 (4)	0.0239 (16)	
C41	0.6997 (7)	0.5020 (3)	0.4281 (4)	0.0223 (14)	
C51	0.5478 (6)	0.4342 (3)	0.4358 (4)	0.0222 (16)	
C61	0.6189 (6)	0.3623 (3)	0.5057 (4)	0.0202 (14)	
O11A	1.1135 (5)	0.52662 (18)	0.1963 (3)	0.0307 (11)	
O12A	0.7721 (5)	0.59140 (19)	0.1352 (3)	0.0338 (11)	
O31A	0.6393 (5)	0.7561 (2)	−0.2381 (3)	0.0411 (11)	
O32A	0.8367 (5)	0.7329 (2)	−0.3913 (3)	0.0568 (14)	
O51A	1.5248 (5)	0.5584 (2)	−0.3210 (3)	0.0452 (11)	
O52A	1.5821 (5)	0.4790 (2)	−0.1557 (4)	0.0518 (14)	
N31A	0.7989 (6)	0.7217 (2)	−0.2837 (4)	0.0304 (12)	
N51A	1.4771 (6)	0.5348 (2)	−0.2196 (4)	0.0302 (14)	
C1A	1.0392 (6)	0.5909 (2)	−0.0076 (4)	0.0202 (14)	
C2A	0.8998 (7)	0.6454 (3)	−0.0849 (4)	0.0227 (14)	
C3A	0.9516 (6)	0.6645 (3)	−0.2025 (4)	0.0228 (14)	
C4A	1.1434 (7)	0.6304 (3)	−0.2487 (4)	0.0237 (14)	
C5A	1.2790 (6)	0.5752 (3)	−0.1701 (4)	0.0205 (14)	
C6A	1.2350 (6)	0.5561 (3)	−0.0500 (4)	0.0234 (14)	
C11A	0.9811 (7)	0.5666 (3)	0.1197 (4)	0.0267 (17)	
H2	1.19500	0.35230	0.85710	0.0260*	
H3	1.14970	0.38670	1.05900	0.0280*	
H5	0.52150	0.27540	1.01240	0.0300*	
H6	0.56660	0.24200	0.80910	0.0280*	
H21	1.15000	0.41890	0.59660	0.0260*	
H31	1.03330	0.53890	0.48110	0.0290*	
H51	0.39760	0.43720	0.39380	0.0270*	
H61	0.51570	0.31740	0.51140	0.0240*	
H411	0.92050	0.36670	1.23450	0.0450*	
H412	0.65340	0.36650	1.20020	0.0450*	
H413	0.48370	0.57210	0.32450	0.0360*	
H414	0.64420	0.62220	0.41090	0.0360*	
H2A	0.76960	0.66950	−0.05720	0.0280*	
H4A	1.17850	0.64400	−0.32840	0.0280*	
H6A	1.33460	0.52070	0.00190	0.0270*	
H12A	0.73000	0.58250	0.21450	0.0510*	

### Atomic displacement parameters (Å^2^)

**Table:**

	*U*^11^	*U*^22^	*U*^33^	*U*^12^	*U*^13^	*U*^23^
S1	0.0254 (5)	0.0146 (5)	0.0240 (6)	0.0027 (5)	0.0059 (4)	0.0007 (5)
O1	0.0231 (14)	0.0290 (17)	0.0322 (17)	0.0077 (14)	0.0084 (11)	0.0032 (15)
O11	0.0409 (17)	0.0127 (15)	0.0319 (18)	−0.0020 (13)	0.0091 (13)	−0.0017 (13)
N4	0.043 (2)	0.045 (3)	0.027 (2)	−0.0099 (19)	0.0168 (18)	−0.005 (2)
N41	0.027 (2)	0.035 (2)	0.030 (2)	0.0116 (16)	0.0147 (16)	0.0147 (18)
C1	0.024 (2)	0.015 (2)	0.021 (2)	0.0046 (16)	0.0057 (17)	0.0073 (17)
C2	0.019 (2)	0.018 (2)	0.028 (3)	−0.0006 (17)	0.0062 (18)	0.0050 (19)
C3	0.024 (2)	0.021 (2)	0.026 (3)	−0.0070 (18)	0.0063 (18)	0.002 (2)
C4	0.031 (2)	0.014 (2)	0.029 (3)	0.0027 (19)	0.0075 (19)	0.005 (2)
C5	0.019 (2)	0.028 (3)	0.029 (3)	0.0011 (17)	0.0065 (18)	0.006 (2)
C6	0.020 (2)	0.022 (2)	0.027 (3)	−0.001 (2)	0.0008 (16)	0.002 (2)
C11	0.020 (2)	0.017 (2)	0.018 (2)	0.0024 (17)	0.0071 (17)	−0.0014 (18)
C21	0.019 (2)	0.019 (2)	0.027 (3)	0.0022 (18)	0.0036 (17)	−0.002 (2)
C31	0.027 (3)	0.015 (2)	0.032 (3)	−0.0054 (18)	0.0119 (19)	−0.002 (2)
C41	0.028 (2)	0.022 (2)	0.020 (3)	0.0052 (19)	0.0144 (18)	0.0014 (19)
C51	0.013 (2)	0.034 (3)	0.020 (3)	−0.0010 (19)	0.0037 (16)	0.001 (2)
C61	0.018 (2)	0.016 (2)	0.027 (3)	−0.0033 (17)	0.0043 (17)	0.0019 (19)
O11A	0.0358 (18)	0.032 (2)	0.0237 (19)	0.0036 (15)	0.0015 (14)	0.0046 (15)
O12A	0.0348 (18)	0.043 (2)	0.0270 (19)	0.0126 (15)	0.0162 (14)	0.0109 (16)
O31A	0.0382 (17)	0.051 (2)	0.036 (2)	0.0219 (18)	0.0123 (14)	0.0015 (19)
O32A	0.050 (2)	0.094 (3)	0.029 (2)	0.0285 (19)	0.0145 (16)	0.023 (2)
O51A	0.0420 (19)	0.058 (2)	0.040 (2)	0.0061 (16)	0.0217 (16)	−0.0068 (17)
O52A	0.040 (2)	0.038 (2)	0.081 (3)	0.0210 (18)	0.0219 (18)	0.011 (2)
N31A	0.032 (2)	0.035 (2)	0.025 (2)	0.0044 (17)	0.0070 (18)	0.0080 (19)
N51A	0.023 (2)	0.025 (2)	0.044 (3)	0.0027 (17)	0.0102 (18)	−0.011 (2)
C1A	0.026 (2)	0.007 (2)	0.028 (3)	−0.0041 (17)	0.0047 (18)	−0.0044 (19)
C2A	0.020 (2)	0.016 (2)	0.031 (3)	−0.0019 (17)	−0.0003 (18)	−0.0017 (19)
C3A	0.019 (2)	0.020 (2)	0.030 (3)	−0.0012 (17)	0.0058 (18)	−0.006 (2)
C4A	0.028 (2)	0.023 (2)	0.021 (3)	−0.0062 (19)	0.0071 (19)	−0.0041 (19)
C5A	0.022 (2)	0.018 (2)	0.022 (3)	0.0016 (17)	0.0050 (17)	−0.0022 (19)
C6A	0.024 (2)	0.016 (2)	0.031 (3)	−0.0009 (19)	0.0068 (19)	−0.002 (2)
C11A	0.033 (3)	0.020 (3)	0.027 (3)	−0.004 (2)	0.004 (2)	−0.002 (2)

### Geometric parameters (Å, º)

**Table:**

S1—O1	1.446 (3)	C11—C61	1.378 (5)
S1—O11	1.439 (3)	C11—C21	1.393 (5)
S1—C1	1.758 (4)	C21—C31	1.368 (6)
S1—C11	1.760 (4)	C31—C41	1.404 (6)
O11A—C11A	1.220 (5)	C41—C51	1.388 (6)
O12A—C11A	1.309 (5)	C51—C61	1.382 (6)
O31A—N31A	1.230 (5)	C2—H2	0.9300
O32A—N31A	1.217 (5)	C3—H3	0.9300
O51A—N51A	1.215 (5)	C5—H5	0.9300
O52A—N51A	1.220 (5)	C6—H6	0.9300
O12A—H12A	0.9300	C21—H21	0.9300
N4—C4	1.357 (5)	C31—H31	0.9300
N41—C41	1.415 (6)	C51—H51	0.9300
N4—H412	0.9500	C61—H61	0.9300
N4—H411	0.8500	C1A—C6A	1.393 (5)
N41—H413	0.8900	C1A—C11A	1.499 (6)
N41—H414	0.8900	C1A—C2A	1.374 (6)
N31A—C3A	1.462 (6)	C2A—C3A	1.369 (6)
N51A—C5A	1.474 (5)	C3A—C4A	1.387 (6)
C1—C2	1.390 (5)	C4A—C5A	1.378 (6)
C1—C6	1.387 (5)	C5A—C6A	1.380 (6)
C2—C3	1.362 (6)	C2A—H2A	0.9300
C3—C4	1.409 (6)	C4A—H4A	0.9300
C4—C5	1.397 (6)	C6A—H6A	0.9300
C5—C6	1.371 (6)		
			
O1—S1—O11	118.73 (18)	C11—C61—C51	120.3 (4)
O1—S1—C1	106.80 (16)	C1—C2—H2	120.00
O1—S1—C11	107.70 (17)	C3—C2—H2	120.00
O11—S1—C1	108.88 (18)	C4—C3—H3	119.00
O11—S1—C11	108.03 (18)	C2—C3—H3	119.00
C1—S1—C11	106.02 (18)	C4—C5—H5	119.00
C11A—O12A—H12A	116.00	C6—C5—H5	119.00
H411—N4—H412	119.00	C1—C6—H6	120.00
C4—N4—H411	122.00	C5—C6—H6	120.00
C4—N4—H412	118.00	C11—C21—H21	120.00
C41—N41—H413	110.00	C31—C21—H21	120.00
C41—N41—H414	109.00	C21—C31—H31	120.00
H413—N41—H414	109.00	C41—C31—H31	120.00
O31A—N31A—O32A	123.9 (4)	C41—C51—H51	120.00
O31A—N31A—C3A	117.4 (4)	C61—C51—H51	120.00
O32A—N31A—C3A	118.7 (3)	C11—C61—H61	120.00
O52A—N51A—C5A	117.4 (4)	C51—C61—H61	120.00
O51A—N51A—O52A	124.2 (4)	C2A—C1A—C11A	121.3 (3)
O51A—N51A—C5A	118.5 (3)	C6A—C1A—C11A	119.6 (3)
S1—C1—C2	118.7 (3)	C2A—C1A—C6A	119.1 (4)
C2—C1—C6	119.7 (4)	C1A—C2A—C3A	120.4 (4)
S1—C1—C6	121.7 (3)	N31A—C3A—C2A	119.4 (4)
C1—C2—C3	120.2 (4)	C2A—C3A—C4A	122.2 (4)
C2—C3—C4	121.3 (4)	N31A—C3A—C4A	118.4 (4)
N4—C4—C5	122.2 (4)	C3A—C4A—C5A	116.3 (4)
C3—C4—C5	117.4 (4)	N51A—C5A—C6A	119.8 (4)
N4—C4—C3	120.5 (4)	C4A—C5A—C6A	123.0 (4)
C4—C5—C6	121.5 (4)	N51A—C5A—C4A	117.3 (4)
C1—C6—C5	119.9 (4)	C1A—C6A—C5A	118.9 (4)
C21—C11—C61	120.1 (4)	O11A—C11A—C1A	122.9 (4)
S1—C11—C21	119.5 (3)	O12A—C11A—C1A	111.6 (4)
S1—C11—C61	120.4 (3)	O11A—C11A—O12A	125.4 (4)
C11—C21—C31	119.8 (4)	C1A—C2A—H2A	120.00
C21—C31—C41	120.6 (4)	C3A—C2A—H2A	120.00
N41—C41—C51	121.6 (4)	C3A—C4A—H4A	122.00
C31—C41—C51	118.9 (4)	C5A—C4A—H4A	122.00
N41—C41—C31	119.5 (4)	C1A—C6A—H6A	121.00
C41—C51—C61	120.3 (4)	C5A—C6A—H6A	121.00
			
O1—S1—C1—C2	29.6 (3)	C3—C4—C5—C6	−0.7 (6)
O1—S1—C1—C6	−152.0 (3)	C4—C5—C6—C1	1.5 (6)
O11—S1—C1—C2	158.9 (3)	S1—C11—C21—C31	−178.4 (3)
O11—S1—C1—C6	−22.7 (4)	C61—C11—C21—C31	1.4 (6)
C11—S1—C1—C2	−85.1 (3)	C21—C11—C61—C51	−1.1 (6)
C11—S1—C1—C6	93.3 (3)	S1—C11—C61—C51	178.7 (3)
O1—S1—C11—C21	−27.7 (4)	C11—C21—C31—C41	0.4 (6)
O1—S1—C11—C61	152.6 (3)	C21—C31—C41—N41	179.0 (4)
O11—S1—C11—C21	−157.0 (3)	C21—C31—C41—C51	−2.3 (6)
O11—S1—C11—C61	23.2 (4)	C31—C41—C51—C61	2.6 (6)
C1—S1—C11—C21	86.4 (3)	N41—C41—C51—C61	−178.7 (4)
C1—S1—C11—C61	−93.4 (4)	C41—C51—C61—C11	−0.9 (6)
O32A—N31A—C3A—C2A	−174.4 (4)	C6A—C1A—C2A—C3A	0.7 (6)
O32A—N31A—C3A—C4A	4.7 (6)	C11A—C1A—C2A—C3A	−177.8 (4)
O31A—N31A—C3A—C2A	6.0 (6)	C2A—C1A—C6A—C5A	−2.1 (6)
O31A—N31A—C3A—C4A	−175.0 (4)	C11A—C1A—C6A—C5A	176.3 (4)
O52A—N51A—C5A—C4A	−172.2 (4)	C2A—C1A—C11A—O11A	−171.3 (4)
O51A—N51A—C5A—C4A	7.3 (6)	C2A—C1A—C11A—O12A	10.5 (6)
O51A—N51A—C5A—C6A	−173.5 (4)	C6A—C1A—C11A—O11A	10.3 (6)
O52A—N51A—C5A—C6A	7.0 (6)	C6A—C1A—C11A—O12A	−167.9 (4)
S1—C1—C2—C3	−180.0 (3)	C1A—C2A—C3A—N31A	179.2 (4)
C6—C1—C2—C3	1.6 (6)	C1A—C2A—C3A—C4A	0.1 (7)
C2—C1—C6—C5	−1.9 (6)	N31A—C3A—C4A—C5A	−178.4 (4)
S1—C1—C6—C5	179.7 (3)	C2A—C3A—C4A—C5A	0.6 (7)
C1—C2—C3—C4	−0.9 (6)	C3A—C4A—C5A—N51A	177.0 (4)
C2—C3—C4—N4	178.9 (4)	C3A—C4A—C5A—C6A	−2.2 (7)
C2—C3—C4—C5	0.5 (6)	N51A—C5A—C6A—C1A	−176.2 (4)
N4—C4—C5—C6	−179.1 (4)	C4A—C5A—C6A—C1A	3.0 (7)

### Hydrogen-bond geometry (Å, º)

**Table:**

*D*—H···*A*	*D*—H	H···*A*	*D*···*A*	*D*—H···*A*
O12*A*—H12*A*···N41	0.93	1.73	2.653 (5)	173
N4—H412···O31*A*^i^	0.95	2.49	3.150 (5)	126
N41—H413···O11*A*^ii^	0.89	2.50	3.367 (5)	165
N41—H414···O1^iii^	0.89	2.50	3.030 (4)	119
C2—H2···O1	0.93	2.58	2.924 (5)	102

Symmetry codes: (i) −*x*+1, *y*−1/2, −*z*+1; (ii) *x*−1, *y*, *z*; (iii) −*x*+2, *y*+1/2, −*z*+1.
